# Sleep and Its Disorders.—I

**Published:** 1895-11-30

**Authors:** Tom R. Taylor


### Medical Progress and Hospital Clinics.

[*The Editor will be glad to receive offers of co-operation a ·d contributions from members of the profession. All letters should be addressed to* The Editor, at the Office, 428, Strand, London, W.C.]

### SLEEP AND ITS DISORDERS.—I.

By Tom R. Taylor, M.D.Lond., B.Sc., F.R.C.S.

Sleep may be regarded as the anabolic or reconstructive phase in cerebral activity. Many of its phenomena have their analogues in the physiological fatigue of other tissues. But, on the other hand, psychical forces having so great an influence upon the more purely somatic side of life, and being equally dependent upon bodily states, it follows that the analogy cannot be pressed too far. During sleep the mental faculties of knowing, willing, and feeling are in abeyance *pro tempore*. Those regions of the brain, however, merely concerned in trophic and the other purely organic functions continue in a state of activity somewhat below the waking degree.

With regard to the senses, they may be said to sleep in different degrees of soundness, taste and smell being heavy sleepers, whilst tactile sense and hearing act like watch-dogs to the slumbering brain. Digestion and assimilation, together with the metabolic processes, all sink to a low ebb. But there are degrees of sleep. There is the sound healthy sleep, waking from which all is blank. There are dreams of varying degrees of vividness and coherency, of which a distinct recollection remains, with impressions as keen and realistic at the time as those of objective life. There is the sleep with an indistinct feeling on waking of having had some experience too vague and intangible to describe, or which may be "broken" later on in the day. And there is that delicious but pernicious state called dozing. In this condition there is some knowledge and feeling, but the highest functions of will are, as we feel it, allowed for a while to lapse, whilst we mentally drift along, party forming ideas, partly experiencing hallucinations which we know at the time to be merely dreams.

The late Mr. Arthur Durham, from his direct experiments upon the brain, found that during sleep there was a diminished blood supply. This he attributed to the wearied brain not inhibiting the ever active vaso-motor centre. It has been suggested, and with reason, that sleep, like the fatigue of muscle, is due not only to the using up of the potential energy, but also to the accumulation of the waste products of nervous activity. Consciousness is the sum total of peripheral stimuli. Imagine all such removed, and it is difficult—in fact, impossible—to realise existence. If no stimuli are received, or if the centres be too depressed to appreciate, then there is no consciousness. All know the greater tendency to sleep when external stimuli are, as far as possible, shut out. Strumpell records an extreme example of this. A boy who had all the sensory inlets paralysed but one eye and one ear, fell asleep when these, his only communications with the objective world, were closed.

The disorders of sleep are numerous. The first and most important one from its frequency, and from the fact that the physician is so often consulted upon the matter, is insomnia. This amounts almost to a disease and causes extreme suffering. It may be subdivided into what may be called, for want of a better title, true insomnia and symptomatic insomnia. Symptomatic insomnia is well known in the sleeplessness occuring in febrile states, in uræmic conditions, and more especially in nervous and mental diseases. It is a prominent symptom and general forerunner in almost all forms of mental aberration. In delirium tremens, for example, the sleeplessness of the patient is most striking. But perhaps the most remarkable cases occur in those states of mental alienation where the patient, hour after hour, with monotonous regularity, repeats some act or phrase, apparently tireless as an automaton.

The treatment of symptomatic insomnia must, if possible, be directed to the "fons et origo mali." Febrile insomnia passes away when the temperature is abated either naturally or artificially. Sponging the surface of the body of such a patient with tepid water will often secure a good night's rest. Antipyretic drugs often have the same effect. In some cases the administration of a little alcohol will act in a most beneficial manner. The use of hypnotics may be necessary in any form of this trouble, but it is more especially in the nervous and mental cases that their use is indicated. Here very large doses may be given, and too often with but little result. Grain-doses of morphia hypodermically, sixty to ninety grains of chloral, &c., are often administered with impunity. In a case of my own, where acute mania with delusions followed the removal of a cystic kidney in a highly neurotic woman, two-thirds of a grain of morphia hypodermically only induced one hour's sleep. The stronger hypnotics, such as hyoscine, should be reserved for these cases. Hydriodate of hyoscine is, perhaps, the most powerful sleep-producing agent. A dose of one seventy-fifth of a grain placed under the tongue in one case induced profound sleep for eighteen hours. It was impossible to wake the patient, who, up to the time of administration, had been noisy and restless. He was suffering from sub-acute mania with delusions and had also epitheliomatous cervical glands. Of milder drugs, sulphonal and chloralamide are safe and valuable agents. A combination of chloral and bromide of potassium may be given as a draught, but should never be persisted in for any length of time.

With regard to what I have styled true insomnia, let us take first of all that of children. Restless, sleepless nights, apart from any definite disorders, are commoner among young children than might at first be supposed. The sufferers, as a rule, are neurotic, precocious youngsters of an imaginative turn of mind. And let us bear this in mind: children know more, think more, and are not nearly so simple-minded as many of us think. A trip to a pantomime, a children's party, or any form of childish dissipation may set one of these highly-strung minds at work. Every incident of the event firmly impressed on the susceptible cortex in all its details is recalled vividly when the association processes are initiated, as they may be by the slightest thing. When the child retires to bed every melody, every song is heard over again, every face is seen, every feeling experienced, and sleep is impossible for hours, until the wearied brain yields, choked, as it were, with its own waste products. In the morning the little one is weary, languid, and peevish. Such cases are not uncommon, but are rarely brought for advice. We would not, if we could, change the temperament, for such children enjoy as they suffer—keenly. But we can adapt life to such, for their life's surroundings are in our hands. No imaginative, eager child should be encouraged to think. The mental side should not be forced. In fact, we may neglect it in our case, for it will develop only too rapidly. Encourage the physical, the purely animal side of life—the love of nature and open air exercise. Let all evening meals be light, and at least two hours before bed-time; and despite protestations and tears, if a *fête* be necessary or desirable, let it be an early one. The insomnia of adult life, like that life itself, is more complicated. There is a form we often see. It occurs in what I may call the superior person. He has some amount of education, and has vast pride in the possession. His occupation is almost reflex, yet nervously exhausting, say a compositor or a clerk. His relaxations are attempts at acquiring knowledge, futile attempts at learning a language by means of Professor X.'s five minutes' method, or a science whose rudiments are skipped, and the great questions of life or the creation are discussed as though they were absolutely puerile by the side of the multiplication table. His mode of life is ascetic. He is a teetotaler and a non-smoker. In fact, all those frivolities which make life what it is—livable to a rational man—are taboo. He cannot sleep at night; hour after hour he tosses about, thinking out great schemes, as he deems them, for the regeneration of mankind, until this sleepless time becomes a habit, and he seeks advice. The sooner such a man realises that he is neither a budding Müller nor a coming Newton, the better for him. He should take to heart Dr. Pascal's motto:—

Connais la vie, aime la, vis la comme elle doit être vécue.

The whole of such a one's mode of life should be gone over in detail and corrected. Healthy relaxation for both mind and body must be provided for; and at first some mild hypnotic may be given an hour before bedtime. Care must be taken, however, if possible, not to let the patient know that he is taking anything of this nature, or at any rate, the particular drug selected. For these men are, as a rule, essentially neurotic, and readily acquire habits.

Then there is the form of insomnia due to overtaxing of the brain, which, like a sorely-tried muscle, quivers as it were when the effort is over—hard-reading men who work till the small hours of the morning, and then immediately go to bed They should devote at least an hour before retiring for the night to resting their minds by reading some literature of a most unexciting nature, and quite foreign to that which they have been perusing. Or they may play cards, or chat, or have a stroll; in fact, anything to divert their thoughts for some time. Those much concerned with figures, *e.g.*, contractors, are often very much troubled by sleeplessness, and this must likewise be attributed to an over-goaded cortex. Chloralamide in a glass of whisky and water before going to bed is a most excellent hypnotic, and should always be given when, as in the case of a harassed business man, a good night's rest has not been obtained for some time and the stress is only likely to be temporary. But if it be found that the strain is constant, then the drug must not be persevered with. The patient must be advised, if he values health and life, to change his pursuit, or, at any rate, find means to lighten the burden of it.

In elderly people, in addition to the preceding forms, there is one we are often consulted about, viz., sleeplessness for some time after going to bed and wakefulness after some early hour in the morning. It must be remembered, however, that afternoon naps are a common luxury amongst those who have borne the heat and burden of the day; and that the twilight of life brings with it diminished activity, and hence less fatigue and necessity for rest.

One cannot leave the subject of insomnia and its treatment without alluding to that infant, as yet, in therapeutics—hypnotic suggestion. Much can be done in the way of suggestion without actually hypnotizing the patient. The following case occurred in the practice of a friend. A lady, a victim to insomnia and its too frequent sequela, the morphia habit, came for advice. She was directed to take every night a small pill, after doing which she was assured she would fall asleep in twenty minutes. The pill was an ordinary dinner pill, but so strongly impressed was she, that the desired result was obtained. She had a good night's rest. Gradually the pills were omitted, and the morphia habit and insomnia were cured. The method of suggestion as practised by the Nancy School has produced most brilliant results in some extremely obstinate cases. At present there are obvious difficulties in its general application. But when a little more of what is now generally regarded as either charlatanism or clever conjuring is known, I have not the slightest doubt that hynotism will take premier place, not only in the treatment of insomnia but of all neuroses—more especially those of functional origin.

